# Performance evaluation and codal assessment of double-skinned solid-core CFST columns with varying steel configurations

**DOI:** 10.1038/s41598-026-45278-7

**Published:** 2026-03-21

**Authors:** Prakhash Neelamegam, S Kanchidurai, V Ganga, Missgna Addisalem Berhe, A. Suganya

**Affiliations:** 1 Department of Civil Engineering, School of Engineering, SR University, Warangal, 506371 Telangana India; 2 Sastra Deemed University, Thanjavur, Tamil Nadu India; 3 Department of Civil Engineering, Thiagarajar College of Engineering, Madurai, 625015 Tamil Nadu India; 4 Department of Civil Engineering, Adigrat University, Adigrat, Ethiopia; 5 Sastra Deemed University, Thanjavur, Tamil Nadu India

**Keywords:** Double skin CFST, Compressive strength, Ductility, Stiffness, Codal comparison, ANN, Engineering, Materials science

## Abstract

This experimental study explores the behaviour of columns with double-skinned solid core concrete-filled steel tubular (DS-CFST) sections. Many experimental studies have been conducted on double-skinned hollow CFST sections so far, and this research has involved evaluating the performance of various configurations of steel tube geometry (square and circular combinations) of solid-core CFST specimens. Eight CFST short columns were subjected to axial compression, each measuring 410 mm in height (H). The study investigated the effects of various parameters, including concrete strength, steel area, width-to-thickness ratio, steel and concrete core percentage, and inner steel embedment position, on CFST short columns with slenderness ratios (λ) ranging from 9.4 to 10.9. The test results showed improvements in DS-CFST column axial compression, ductility, stiffness, failure modes, and structural behaviour due to adequate steel inner tube embedment. The CFSC specimens exhibited 6.64 times higher results than the steel tubes and 2.33 times greater strength than the CFST specimens, while other steel tube embedded specimens demonstrated significant strength improvements. The results are checked with the current codal provisions, ANSI/AISC-360, EC-4 and modelled by an artificial neural network (ANN).

## Introduction

Concrete-Filled Steel Tubes (CFSTs) have emerged as a revolutionary structural system in modern construction, offering a superior alternative to traditional reinforced concrete columns. These composite members demonstrate enhanced load-bearing capacity, often exceeding that of reinforced concrete columns by a factor of three, owing to the synergistic interaction between the steel tube and the confined concrete core. Among the various CFST innovations, Double-Skin Concrete-Filled Steel Tubes (DS-CFSTs) have attracted considerable attention due to their superior structural performance and serviceability under demanding conditions.

DS-CFSTs exhibit numerous advantages, including improved ductility, accelerated construction timelines, simplified fabrication, and enhanced architectural flexibility. Moreover, these systems facilitate the design of taller and more slender buildings while offering superior seismic performance, durability, and resistance to sudden failure mechanisms^1–7^. The solid-core design eliminates internal voids, thereby promoting uninterrupted concrete–steel interaction, superior confinement effectiveness, improved stress transfer between the steel tubes and the concrete core, and reduced vulnerability to inward local buckling—a phenomenon prevalent in hollow-core configurations^5^. Moreover, the unique load-transfer mechanisms and failure behaviours of solid-core DS-CFST members demonstrate that design methods formulated for hollow-core or multi-cell CFST systems are not directly transferable. In addition, the principal contributions of this research are now distinctly articulated at the close of the Introduction, encompassing the introduction of an innovative solid-core DS-CFST system, its experimental assessment under axial compression, benchmarking against established CFST variants, and scrutiny of the pertinence of extant design provisions. The square cross-section remains the preferred geometry for CFST columns in high-rise construction due to its ease of connection and architectural integration. Recent advancements have explored the incorporation of internal stiffeners and double-skin configurations, which significantly enhance the axial strength, torsional rigidity, and energy dissipation capacity of CFST columns^8–10^. For instance, implementing a 60% double-skinned configuration has shown notable improvements in the flexural behavior of beam elements. However, an increased hollow ratio can negatively affect confinement efficiency and structural integrity. Consequently, this study adopts a solid-core configuration to ensure optimal compressive strength^11^. Yen et al. ^12^ and subsequent research^13^ investigated the behavior of multi-cavity and stiffened DS-CFST members, emphasizing that parameters such as the hollow ratio and inner strip height substantially influence load-carrying capacity. Wengang et al. ^14^ evaluated 1000 mm-high double-skin CFST columns connected using high-strength friction grip (HSFG) bolts and steel sleeves, incorporating a 4 mm-thick circular outer tube with a 22.3%–44% hollow section. Although this configuration reduced the cross-sectional area, it only moderately decreased the axial compressive strength, highlighting the structural feasibility of hollow-core designs.

Similarly, Li et al. ^15^ compared the performance of solid-core CFSTs to multi-cavity configurations, concluding that solid-core members exhibited higher strength and stiffness^16^. Sulthana et al. ^17–19^ further explored DS-CFST columns with a 4500 mm length, featuring 180 mm square outer tubes and 60 mm inner square or circular tubes. Testing five columns with a slenderness ratio of 86.6 and length-to-diameter ratio of 25, they observed axial capacities ranging from 1400 kN to 1650 kN. Their findings indicated significant performance differences between solid and hollow-core columns, particularly when slenderness ratios exceeded 80 ^18,19^. Although multi-cavity and hollow-core DS-CFST designs offer design and construction benefits, their compressive strength gains are often less pronounced than those achieved by single-skin solid-core configurations^20^. Prior investigations into steel tube-confined concrete columns have demonstrated superior strength, ductility, and energy dissipation under both monotonic and cyclic loading, with circular sections outperforming square ones, and code predictions proving conservatively safe. Previous studies^21–23^ have demonstrated that the axial performance of double-skin concrete-filled steel tubular (DS-CFST) columns is significantly influenced by factors such as confinement effects, geometry, and material configuration. These studies have shown that improvements in both strength and ductility are observed across various CFST and hybrid tubular systems, particularly with respect to the geometry of the steel tube and the interaction between the concrete core and steel tube. Our findings align with these results, as we observed similar enhancements in axial strength and ductility, particularly in specimens with optimized geometries and effective confinement. Alsamawi et al. ^24,25^ investigated experimentally and analysed numerically fully encased composite sections with a focus on the steel–concrete interface and bonding mechanisms. Their study demonstrated that the introduction of shear connectors significantly enhanced composite action, resulting in an improvement in ductility of up to 25% under cyclic loading conditions. In contrast to previously studied hollow-core DS-CFST and multi-cell CFST systems, the proposed solid-core DS-CFST configuration eliminates internal voids, leading to enhanced concrete–steel interaction and improved confinement. This solid-core design not only reduces susceptibility to inward local buckling, a common issue in hollow-core systems, but also ensures superior axial load-bearing capacity and ductility by providing more effective confinement to the concrete core. Additionally, the solid-core configuration enhances the overall mechanical behavior of the column by allowing for a more uniform load transfer between the steel tube and the concrete core, improving stress distribution and energy absorption.

This research aims to experimentally investigate the axial behaviour of Double-Skinned Concrete-Filled Steel Tubular (DS-CFST) columns with solid concrete cores, thereby advancing current understanding beyond existing hollow-core configurations. The study involves eight short CFST specimens, each 410 mm in height, designed with slenderness ratios (λ) ranging from 9.4 to 10.9 to represent typical short-column behaviour. These specimens incorporate different combinations of square and circular outer and inner steel tubes enclosing a solid concrete core. The experimental program evaluates the influence of key parameters such as concrete compressive strength, steel cross-sectional area, width-to-thickness (B/t) ratios, concrete-to-steel volume proportions, and the embedment characteristics of the inner steel tube. The axial behaviour of composite columns is strongly governed by their geometric configuration and material interaction. In particular, the presence of a solid concrete core enhances ductility, confinement, and load-bearing capacity, which are crucial for structural stability. Recent research has extensively explored hollow double-skin CFST sections; however, limited studies have addressed solid-core variants. This research addresses that gap by examining how solid-core arrangements and skin geometry affect the performance of DS-CFST columns. The variations in tube geometry are intended to reflect practical design conditions and optimize structural efficiency. In addition to experimental analysis, the study benchmarks the observed behaviour against established international design codes such as ANSI/AISC 360 and Eurocode 4 (EC4) to validate compliance. A predictive Artificial Neural Network (ANN) model is developed to estimate axial strength based on material and geometric input parameters. This study provides a foundation for improved design strategies for seismic-resistant, high-rise, and sustainable structural systems.

## Materials and methods

### Concrete mix

The CFST composite columns are filled with conventional concrete, the constituents of which are detailed in Table 1. The study utilised Portland pozzolana cement with a certified 43 grade; the cement had a fineness of 350 m^2^/kg and a standard consistency of 30.5%. M-sand with 90% of 1.8 mm particle size and a unit weight of 1670 kg/m^3^ was used in the concrete mix. Additionally, the concrete was incorporated with 10 mm coarse aggregates with a unit weight of 1700 kg/m3 and a compressive strength of 27 MPa. The average compressive strength of concrete cast with a 150 × 150 × 150 mm mould and after 28 days of water curing, the concrete cube was tested as per the guidelines given in IS 456: 2000 ^26^. The concrete mix design was calculated following IS 10262:2019 ^27^, and a constant target grade of 20 MPa was maintained for the DSCFST specimens. The compressive strength test was performed after 28 days of curing, and the mechanical properties were determined using a Universal Hydraulic Testing Machine (UTM-1000) with a capacity of 1000 kN. The average compressive strength of the concrete was reported in Table 2, which corresponds to the design strength of C20.

**Table 1:** Mix proportion of concrete.

Material	Cement	Fine aggregate (F.A)	Coarse aggregate (C.A)	water
Percentage by weight	1	1.45	2.96	0.41

**Table 2:** Compressive strength of concrete.

Specimen ID	Weight of materials in ‘kg’ per m^3^				C/S area of cube in ‘mm^2^’	Maximum Load (*P*) In ‘kN’	Compressive stress of concrete ($\:{f}_{c})$ In ‘*N*/mm^2^’
	Cement	FA	CA	Water			
CS-C-01	410	594.5	1213.6	168.1	22,500	499.4	22.19
CS-C-02	410	594.5	1213.6	168.1	22,500	476.3	21.12
CS-C-03	410	594.5	1213.6	168.1	22,500	507.8	22.57
Average compressive strength of concrete$\:{(f}_{c,avg})$							21.96

**Table 3:** Parameters and label of tested specimens.

Label	Shape		H (mm)	Outer (mm)		$\:{A}_{S,o}$(mm^2^)	Inner (mm)		$\:{A}_{S,i}$(mm^2^)	$\:{A}_{S,t}$(mm^2^)	$\:{A}_{C}$(mm^2^)	$\:{A}_{S,\%}$
	Outer	Inner		$\:{B}_{o}$	$\:{t}_{o}$		$\:{B}_{i}$	$\:{t}_{i}$				
SQT01	Square	–	410	150	2	1168	0	0	0	1168	–	5.19
CIT-02	Circular	–	410	150	2	943	0	0	0	943	–	5.33
CFS-03	Square	–	410	150	2	1168	0	0	0	1168	21,332	5.19
CFC-04	Circular	–	410	150	2	943	0	0	0	943	16,736	5.33
CFSC-05	Square	circular	410	150	2	1168	50	1.6	251	1419	21,081	6.31
CFCS-06	circular	square	410	150	2	943	50	1.4	264	1207	16,472	6.83
CFSS-07	Square	square	410	150	2	1168	50	1.4	264	1432	21,068	6.36
CFCC-08	Circular	Circular	410	150	2	943	50	1.6	251	1194	16,485	6.75

### Outer/inner steel tube geometry and material properties

The composite specimens were designed with varied steel tube configurations to investigate the influence of geometry on axial performance. The outer steel tubes were fabricated in two distinct geometries: square sections with dimensions of 150 mm × 150 mm and a wall thickness of 2 mm, and circular sections with an outer diameter of 150 mm and a uniform thickness of 2 mm, as referenced in ^28^. The inner steel tubes, positioned concentrically within the outer shell, also varied in geometry—square sections measuring 50 mm × 50 mm with a thickness of 1.6 mm, and circular sections with an outer diameter of 50 mm and a wall thickness of 1.6 mm, as detailed in Table 3. All steel tubes were manufactured using hot-rolled structural steel sourced from a local supplier, possessing a verified average yield strength $\:\left({f}_{y}\right)$ of 310 N/mm². The specimens were designed as short columns, each with an average height (H) of 410 mm. Based on their geometry and height, the calculated slenderness ratios (λ) were approximately 9.47 for specimens with square outer sections and 10.93 for those with circular outer sections. These low slenderness ratios ensure that the specimens predominantly exhibit axial compression behaviour, allowing for reliable investigation into the local confinement effects and load transfer mechanisms between the steel skin and concrete core. This geometric diversity provides critical insight into the role of tube shape and material distribution in DS-CFST column performance.

### Specimen preparation

**Fig. 1:**
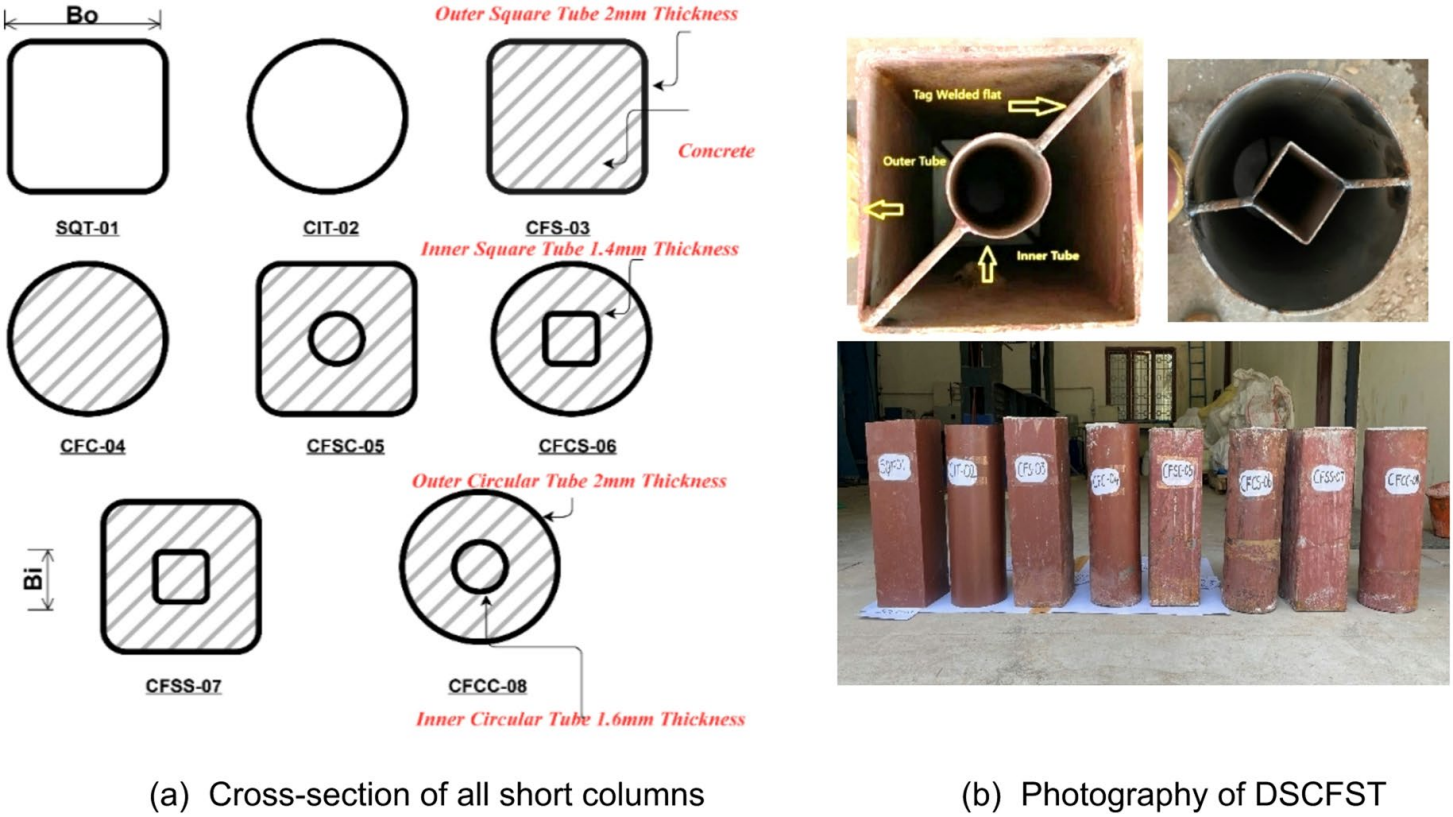
Characteristics of the short column specimen geometries.

This study investigated the axial compressive behaviour of 8 distinct DSCFST (double-skin concrete-filled steel tube) short columns. The specimens comprised the following configurations: 1 square steel tube (SQT-01), one circular tube (CIT-02), one concrete-filled square tube (CFS-03), one concrete-filled circular tube (CFC-04), one concrete-filled square outer and circular inner tube(CFSC-05), one concrete-filled circular outer and square inner tube(CFCS-06), one concrete-filled outer square and inner square tube(CFSS-07), and one concrete-filled outer circle and inner circle tube(CFCC-08). The cross-sectional dimensions of these eight different column specimens are illustrated in Fig. 1(a). The 50 mm x 50 mm square and 50 mm circular inner tube ($\:{B}_{i}$Specimens were utilised to fabricate the DSCFST specimens. The width of the square outer specimen ($\:{B}_{o}$) was 150 mm, and the outer diameter was also 150 mm. The thickness ($\:{t}_{o})$The outer specimen was 2 mm, while the thickness ($\:{t}_{i})$The inner specimen was also 1.4 and 1.6 mm, as specified in Table 3. The overall height of the short column was consistently maintained at 410 mm. The length-to-width ratio (L/D) was kept at 2.73. Figure 1(b) shows the DSCFST short column cross-section fabrication, and the inner tube was centred and connected with a 16 mm x 3 mm steel flat.

Arc welding is commonly used to form tug-butt (butt) connections for inner steel components used in concrete-filled steel tubular (CFST) members. In CFST construction, inner steel plates, rings, stiffeners, or shear transfer elements are often required to ensure composite action between the steel tube and the infilled concrete.

For CFST inner placement, butt welding ensures full-strength continuity of the steel element without introducing eccentricity or stress concentration. Proper edge preparation, controlled heat input, and adequate weld penetration are essential to avoid defects such as lack of fusion or excessive residual stresses, which may affect composite performance. Once welded, the inner steel assembly acts as an effective load-transfer and confinement mechanism.

The standard arc weld was used for the tacking corners. The CFSS-07 configuration, featuring a square outer tube and square inner tube, exhibited the most significant combined cross-sectional area of steel and concrete. Conversely, the CFCC-08 arrangement, comprising a circular outer tube and a circular inner tube, displayed a relatively diminished combined area of steel and concrete. Once the steel tubes were ready, the bottom of the DSCFST was sealed, and M20 concrete was poured layer by layer with a tamping rod. The concrete was poured at room temperature. The specimens were cured by placing wet gunny bags over the exposed concrete parts at the top and bottom. After 28 days of curing, the concrete surfaces at the top and bottom were ground smooth for testing. Finally, the base and top plates were fixed to ensure uniform loading distribution.

### Experimental set-up and instrumentation

**Fig. 2:**
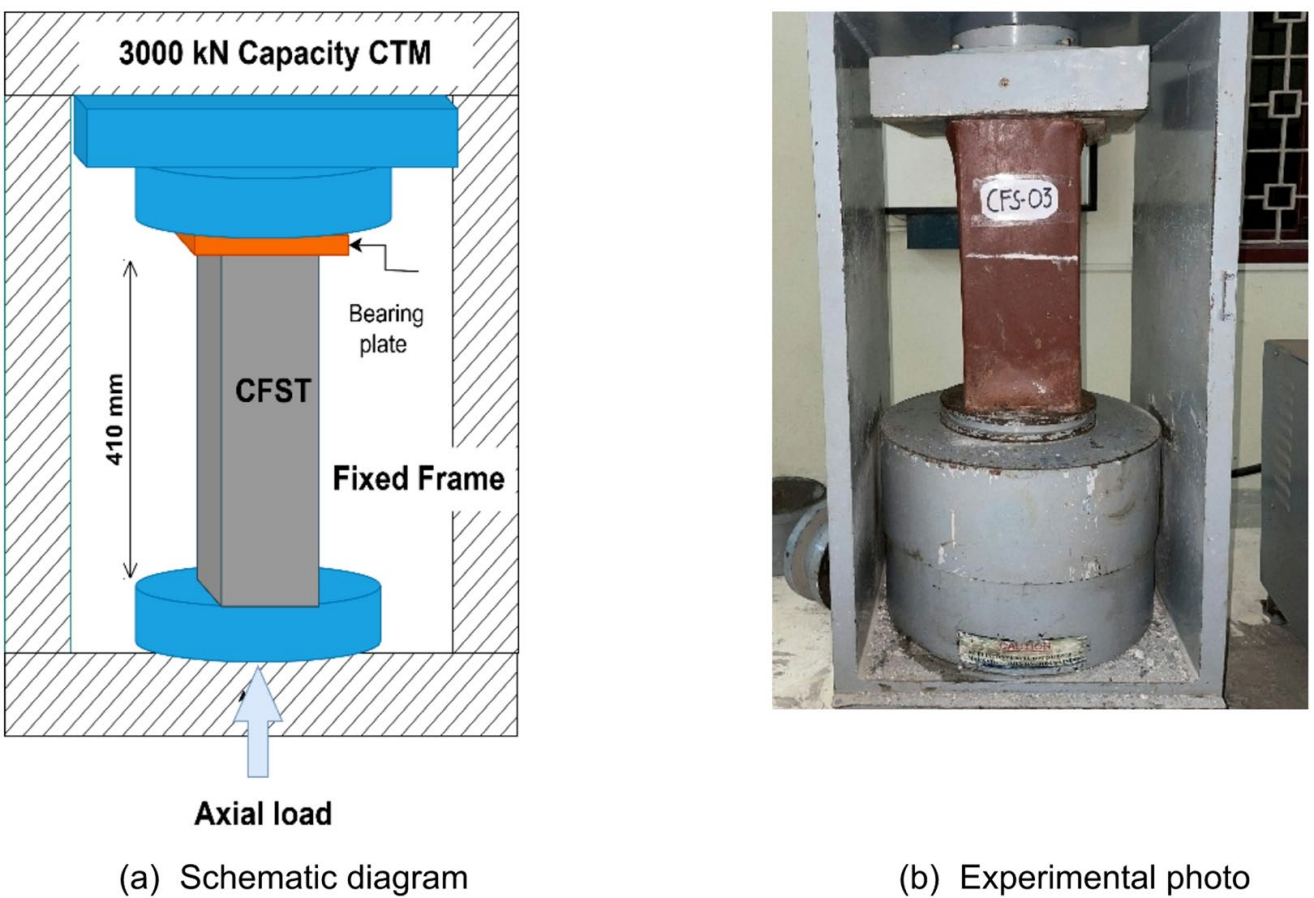
Experimental set-up.

The axial compressive strength of eight CFST specimens was tested using a compression testing machine (CTM) with a 3000 kN loading capacity, as shown in Fig. 2(b). The servo-hydraulic system was computer-controlled and conducted at SASTRA Deemed University. Both ends of each specimen were provided with hinged (pinned) end conditions to simulate realistic boundary conditions and to allow rotation during loading, thereby minimizing the influence of end restraint moments. The specimens were carefully aligned along the loading axis and centrally positioned between the UTM platens to ensure uniform axial load transfer and to reduce eccentricity effects. Axial compression was applied under monotonic loading at a controlled rate until failure, and the corresponding load–deformation behaviour was continuously recorded. During testing, observations were made regarding local buckling of the steel tube, crushing of the infilled concrete, and overall failure modes. The test results obtained were used to evaluate the axial load-carrying capacity, stiffness, and composite action of the CFST specimens. Since the column was in a short category, the maximum compressive strength and the elephant foot bulging, intermediate failure, and shear failure were recorded. The schematic diagram of the test specimen is shown in Fig. 2(a).

## Results and discussion

The failure behavior and distinctive characteristics of the DS-CFST columns were analyzed. The performance of the SQT, CIT, CFS, CFC, and all DS-CFST configurations was evaluated based on axial compressive strength, failure modes, Ductility Index (DI), stiffness (K), and load–deflection response. Furthermore, the influence of the double-skin configuration and the effect of different built-in interface shapes were also discussed. It should be noted that only one specimen was tested for each configuration in this study, which limits the ability to assess experimental variability and impacts the robustness of the conclusions drawn. The results presented here are based on the behavior of a single specimen for each configuration, and as such, should be considered preliminary.

### Axial compressive strength

Axial failure in the DSCFST specimens was defined as the point at which either the maximum axial shortening reached 20% of the column height or a distinct reduction in peak load was observed under sustained axial loading. The axial compressive strength $\:{N}_{u,\mathrm{e}\mathrm{x}\mathrm{p}}$ of all specimens was experimentally evaluated, and the results are summarized in Table 4. The concrete core used in all columns exhibited an average compressive strength of 21.96 MPa, while the yield strength of the steel tubes was confirmed to be 310 MPa through material testing. The experimental findings demonstrate a consistent trend, circular cross-section specimens outperformed their square counterparts in terms of axial compressive capacity^29^. For instance, the hollow circular steel tube specimen (CIT-01) achieved 127% higher axial strength than the hollow square section (SQT-01), clearly highlighting the superior confinement and stress distribution capabilities inherent in circular geometry^30^. It should be noted that this threshold is used solely for the purpose of comparison within this study and does not represent a universal failure criterion. Similarly, among the single-skin concrete-filled tubes, the circular specimen (CFC-04) exhibited 40% higher strength than the square counterpart (CFS-03). This performance difference is attributed to the more uniform hoop stress and confinement pressure generated in circular sections, which delays local buckling and enhances post-peak behaviour^31^.

In the double-skin specimens, CFCS-06 outperformed CFSC-05, with a 28% strength advantage, suggesting that a circular outer tube provides a more efficient confinement envelope. Moreover, the CFCC-08 specimen displayed a 22% improvement in compressive strength over CFSS-07. Notably, CFCS-06 and CFCC-08 despite having relatively lower combined steel and concrete cross-sectional areas recorded substantial axial compressive strengths of 1325.8 kN and 1269.7 kN, respectively. This indicates that geometry and confinement efficiency play a more critical role than cross-sectional area alone^32^. The enhanced axial performance observed in circular configurations is primarily due to the favorable stress trajectories and effective lateral confinement provided by the outer tube, especially in specimens with well-embedded inner tubes. The circular geometry facilitates the development of triaxial compressive stress in the concrete core, thereby mobilizing higher concrete strength through confinement effects. Additionally, the circular steel tube offers uniform radial stiffness, which minimizes stress concentrations and delays the onset of local buckling^13^. These mechanisms synergistically contribute to greater energy absorption capacity and ductility under axial loading. These findings confirm that careful selection of cross-sectional geometry—particularly favoring circular outer skins not only improves load-carrying capacity but also enhances stability and deformation capacity in double-skin CFST systems, making them more suitable for high-performance structural applications where axial loading is critical.

**Fig. 3:**
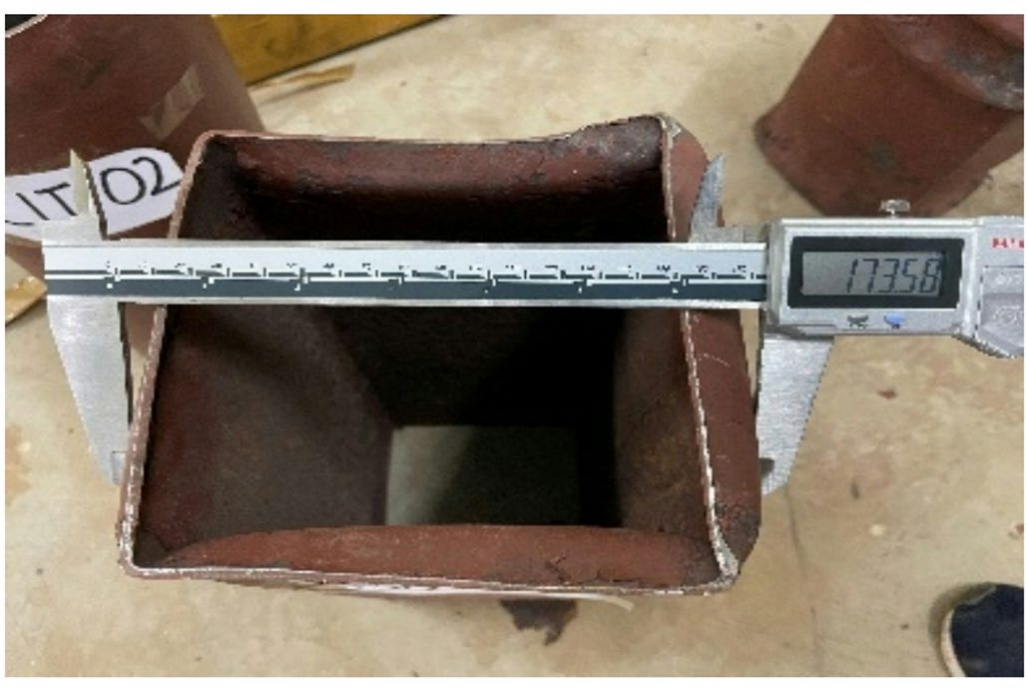
Top view of local buckling of hollow section.

### Failure modes

**Fig. 4:**
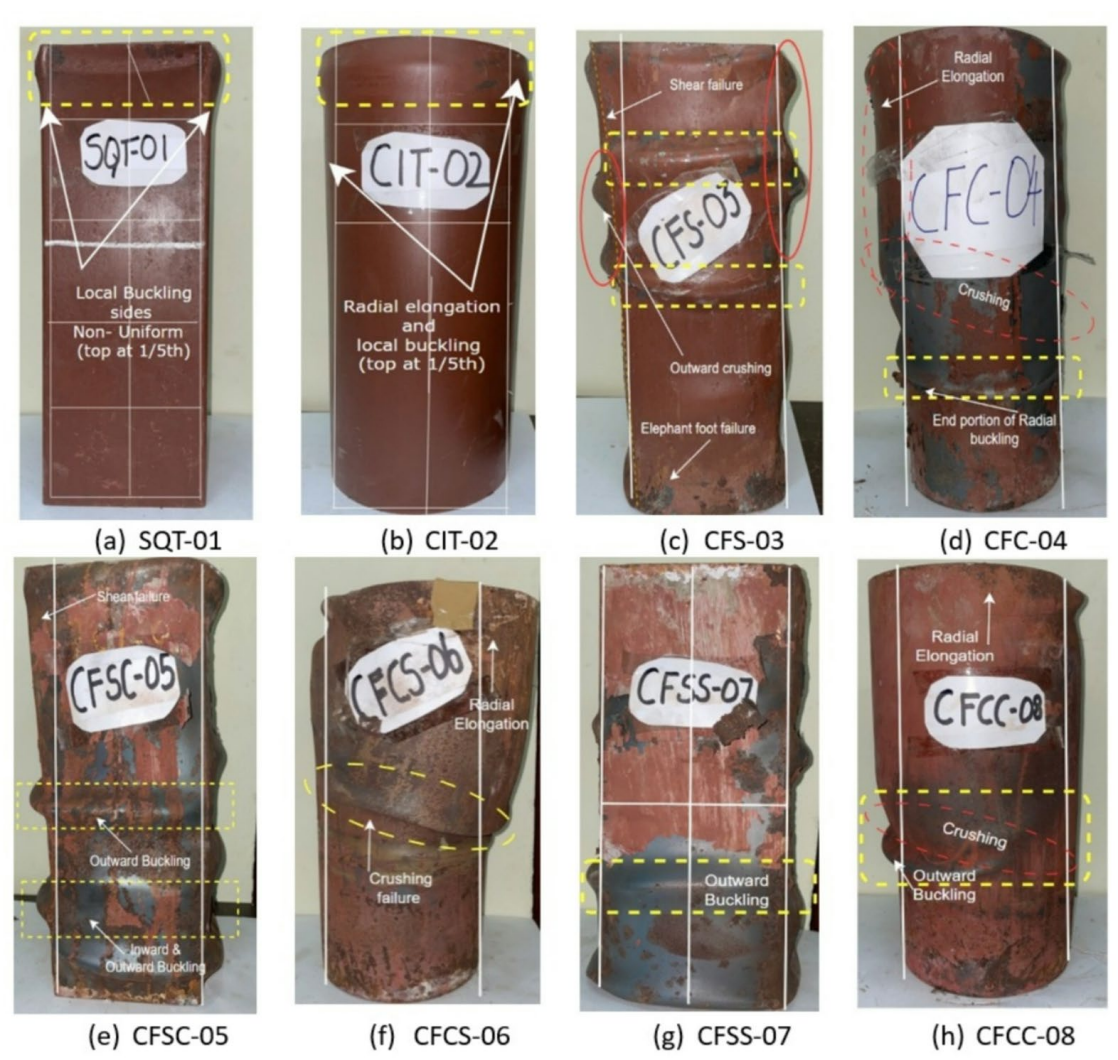
Different failure modes of DS-CFST column specimens.

The comprehensive analysis of how axial compressive force and varying geometric configurations influenced the buckling, shear, and cracking behaviours of CFST and DSCFST specimens is illustrated in Fig. 4. Specimens without concrete infill, namely SQT-01 and CIT-02, exhibited local buckling failure mechanisms. These failures occurred within the top one-fifth region of the specimen height, where the load was directly applied. The absence of a concrete core in these specimens removed the internal restraining effect typically provided by infill, allowing early local instability to occur under axial loading^33^. Post-failure dimensional changes in these hollow specimens highlighted the effect of cross-sectional shape. The square specimen SQT-01 showed non-uniform outward buckling, with one face expanding to 173.8 mm while the opposite side expanded only to 149.8 mm, deviating significantly from the original 150 mm square base. This asymmetric deformation pattern indicates stress concentration along the flat faces and corners, leading to premature and uneven buckling^13^. In contrast, CIT-02, the hollow circular specimen, underwent relatively uniform radial expansion to 172.7 mm from its original 150 mm, suggesting a more stable and symmetric local buckling mechanism due to the uniform stress distribution around the circumference. Figure 3 presents the top view of SQT-01, visually confirming this asymmetric deformation. Axial compression was applied using a displacement-controlled loading method, with deflections ranging from 1 mm to 6 mm across all specimens. This approach enabled accurate identification of the yield and peak load points. During the initial loading phase, all specimens experienced lateral expansion due to Poisson’s effect. However, this expansion exhibited greater variability in the DSCFST specimens, highlighting the influence of inner core confinement and outer geometry^34^.

In the single-skin CFST specimens, the circular configuration again demonstrated superior behaviour. The circular CFST specimen, CFC-04, exhibited higher load-bearing capacity than the square CFST, CFS-03. Moreover, the local failure mode was largely suppressed in both CFC-04 and CFS-03, confirming that concrete infill plays a critical role in restraining outward buckling. However, the square CFS-03 still displayed several undesirable failure features, including “elephant foot” buckling, diagonal crushing, and significant radial elongation from 150 mm to 182 mm. These failure modes were more severe due to stress concentrations along the flat surfaces and corners, which are inherently more prone to localized instability^35^. Distinct failure characteristics were observed among DSCFST specimens based on their geometric configurations. The outer square specimens, CFSC-05 and CFSS-07, exhibited less ductile responses and higher susceptibility to asymmetric deformation compared to the circular outer specimens. In contrast, CFCS-06 and CFCC-08, which had circular outer tubes, displayed controlled radial elongation upon failure. Specifically the outer diameter expanded from 150 mm to 185 mm over the upper three-quarters of the specimen height, while the lower quarter remained relatively unexpanded. This staged deformation behavior suggests that the circular outer tube allowed for a gradual redistribution of hoop stresses, delaying the onset of catastrophic failure and enhancing ductility^36^.

The observed superior performance of circular outer tubes in DSCFST specimens can be specifically attributed to their ability to generate uniform hoop tension and circumferential confinement pressure, which effectively restrains the core concrete and delays outward deformation^37^. Unlike square sections, which have flat sides that tend to bulge outward and corners that act as stress risers, circular tubes offer continuous geometry that facilitates radial pressure without localized weak points. In double-skin configurations, this becomes more critical, as the outer circular tube not only confines the core concrete but also supports the inner tube indirectly through arching action and circumferential stress flow. This dual confinement mechanism is particularly evident in CFCS-06 and CFCC-08, where progressive radial expansion across the specimen height reflected better energy absorption and redistribution of axial stress, culminating in enhanced load-carrying capacity and post-peak stability^38^.

### Ductility index and stiffness

The Double-Skin Concrete-Filled Steel Tube (DS-CFST) short columns exhibited considerable plastic deformation capacity, as captured by the ductility index (DI). This behaviour is especially notable considering the DS-CFST configuration enables the columns to sustain elevated axial loads without premature local buckling. This enhanced stability is primarily attributed to their low slenderness ratios, which improve the axial stiffness and delay instability mechanisms typically seen in thin-walled steel members. The ductility index was derived using Eq. (1), where ∆_(u,0.85) corresponds to the deformation when the post-peak load drops to 85% of the maximum, and ∆_y indicates the deformation at the yield point, defined as the point where the load-displacement curve first deviates from the linear elastic region, marking the onset of plastic deformation in the composite column.^39–42^.

**Fig. 5:**
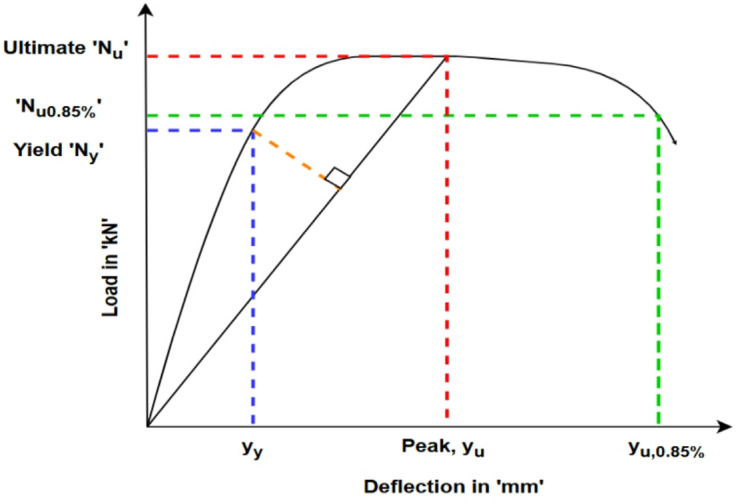
Ductility index key indicator.

1 $$\:D.I=\:\frac{{\varDelta}_{u,\:0.85}}{{\varDelta}_{y}}$$

The DI offers a practical measure of energy dissipation capacity, particularly useful in assessing seismic performance. As illustrated in Fig. 5, the $\:{\varDelta}_{u,\:0.85}\:and\:{\varDelta}_{y}\:$points on the load-deformation curve clearly demonstrate the extended inelastic behaviour exhibited by DS-CFST columns. Higher DI values correlate to better performance in displacement-driven scenarios, typical of earthquake loading. Table 4 presents the calculated ductility indices for the various specimens. While traditional design logic suggests that increasing the steel cross-sectional area enhances ductility, the test results reveal that geometrical configuration plays a more influential role. Notably, the CFCS-06 and CFCC-08 specimens despite having ~ 20% less embedded steel than CFSS-07 exhibited 39.2% and 47.69% higher ductility indices, respectively. This enhanced performance is attributed to the favourable stress redistribution and confinement effects provided by their circular and corrugated geometries^6^. Such configurations likely delay local buckling and promote the formation of stable plastic hinges, contributing to superior post-yield behaviour^30^.

The ductility and initial stiffness was evaluated based on the slope of the elastic segment of the load-deformation curve. As shown in Table 4, the CFCS-06 specimen displayed the highest stiffness value among the configurations. This improved stiffness can be credited to effective load sharing between the concrete core and both the inner and outer steel tubes, which enhances confinement and reduces lateral strain. The stiffness advantage also implies improved load transfer efficiency^43^, which is crucial in applications involving cyclic or repetitive loading. From a local buckling resistance perspective, the presence of the inner steel tube in DS-CFSTs not only improves confinement but also stabilizes the outer shell, effectively mitigating inward or outward buckling under axial loads. This dual-shell system acts as a constraint mechanism, allowing the concrete core to undergo enhanced plastic deformation before failure^44^. The experimental findings also underscore the importance of composite interaction in DS-CFST systems. The energy absorption capacity, inferred from the area under the load-deformation curve, was found to be superior in the CFCS-06 and CFCC-08 specimens. This characteristic is critical for structures subjected to impact or blast loads, where high toughness and ductility are required. In terms of crushing resistance, CFCS-06 demonstrated superior behaviour, with CFC, CIT, and CFCC configurations also showing robust performance. These highlights that circular and corrugated geometries are not only effective for ductility enhancement but also for improving axial strength and post-peak load retention^45^.

**Table 4:** Axial compressive strength and ductility index of tested specimens.

Specimens	$\:{N}_{y,exp}$ ‘kN’	$\:{\varDelta}_{y}$ ‘mm’	$\:{N}_{u,exp}$ ‘kN’	$\:{\varDelta}_{u}$ ‘mm’	$\:{\varDelta}_{u,0.85}$ ‘mm’	DI	Stiffness (K)‘kN/cm’
SQT01	161.02	1.50	174.90	1.15	0.98	0.65	5.96
CIT-02	298.74	2.10	397.50	2.27	1.93	0.92	10.27
CFS-03	759.30	2.40	847.70	4.80	4.08	1.70	29.20
CFC-04	966.17	2.20	1186.80	5.44	4.62	2.10	30.10
CFSC-05	825.27	2.60	1031.60	5.60	4.76	1.83	28.46
CFCS-06	1032.80	2.90	1325.40	10.24	8.70	3.00	35.61
CFSS-07	864.30	3.40	1037.10	8.24	7.00	2.06	25.42
CFCC-08	908.91	3.00	1269.70	9.35	7.95	2.65	30.30
Mean	826.88	2.64	1034.79	6.38	5.64	2.16	25.69
Standard Deviation (SD)	288.47	0.65	246.12	3.60	3.07	0.88	8.59
Coefficient of Variation (CV)	0.35	0.25	0.24	0.57	0.54	0.41	0.33

### Load deflection curves

**Fig. 6:**
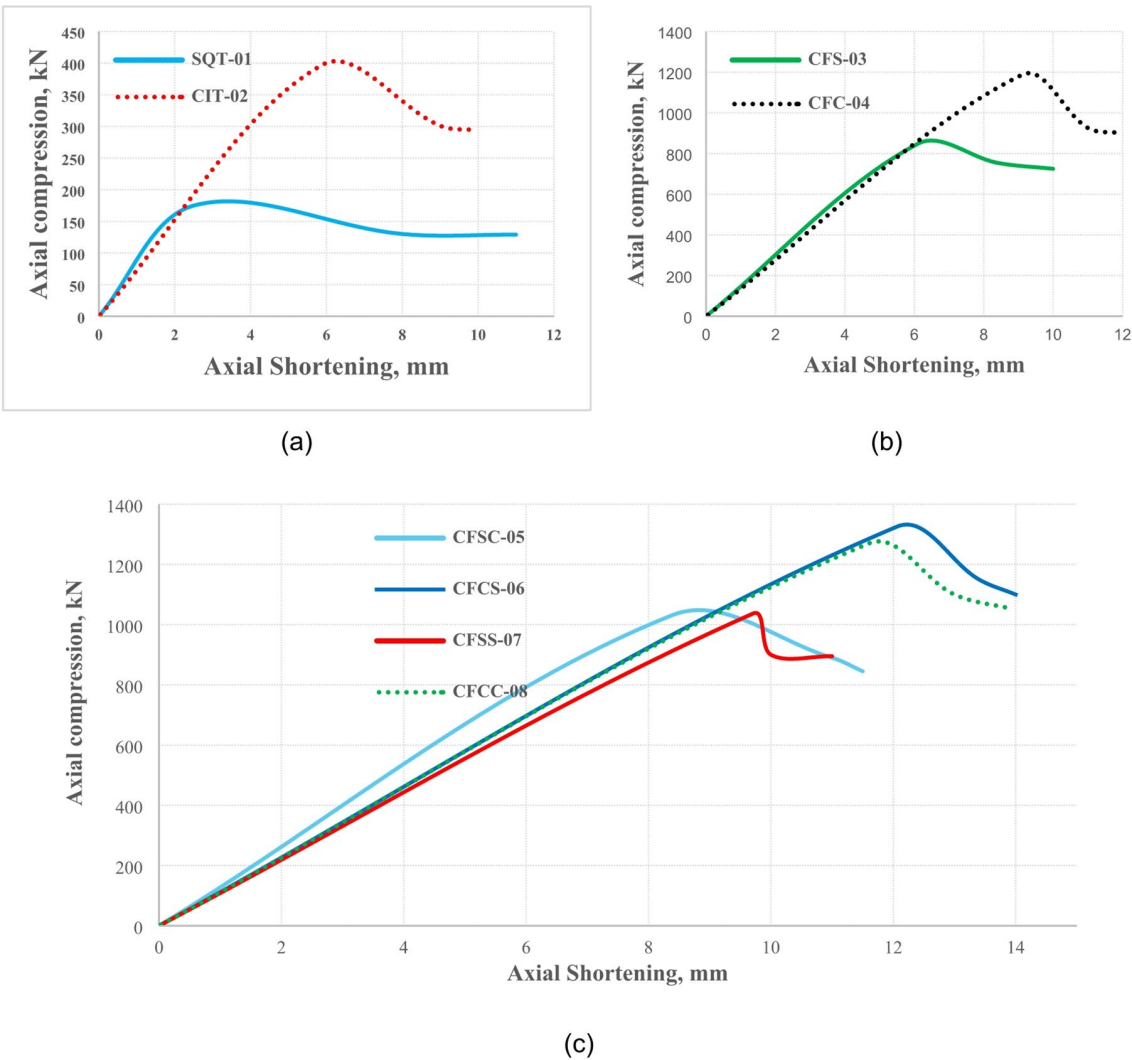
Load Vs Deformation curves of (**a**) Steel tubes, (**b**) CFST and (**c**) DS-CFST solid core.

The axial load–displacement (N–∆) behaviour of the tested specimens was systematically analysed and classified according to their cross-sectional configurations—namely, bare steel tubes, single-skin concrete-filled steel tubes (CFST), and double-skin concrete-filled steel tubes (DS-CFST) incorporating various combinations of inner and outer geometries. As illustrated in Fig. 6, the influence of cross-sectional shape on structural performance was assessed through deformation measurements and evaluated across both elastic and plastic regimes^46,47^. In most cases, complete structural collapse was not observed; instead, several specimens continued to undergo plastic shortening post-peak, indicating sustained load-carrying capacity beyond the elastic limit. The peak or ultimate load was recorded as the failure load for comparative analysis, as this value corresponds to the onset of significant nonlinear behaviour in the N–∆ response. The gradual decline or plateauing of load in the plastic region was associated with strain hardening, local buckling, or progressive concrete crushing, depending on the geometry and confinement effects^48,49^. Axial deformation was manually recorded using linear variable differential transformers (LVDTs), which were strategically placed to capture accurate displacement readings along the specimen’s length. The LVDTs were calibrated prior to testing to ensure precision, and their measurements were continuously monitored during the experiments to capture deformation across both elastic and plastic ranges.

#### Influence of cross-sectional geometry on behaviour

Among the evaluated configurations, square cross-section specimens consistently exhibited lower peak strength and higher deformation under axial loading when compared to their circular counterparts. Specifically, as shown in Fig. 6(a), the SQT-01 specimen (square steel tube only) recorded a peak load of 174.9 kN at an axial displacement of 2.5 mm, and continued to deform until a maximum displacement of 11 mm. The observed failure was characterised by outward local buckling, surface cracking, and coating detachment, indicating poor confinement and stress concentration along the flat faces of the square tube. The absence of internal concrete filling or confinement reinforcement further exacerbated the failure through shear crushing and abrupt loss of stiffness, which is typical for square profiles under axial compression due to stress accumulation at the corners and panel zones^50^. Figure 6(b) highlights the enhanced structural response of circular CFST specimens, such as CFC-04, which reached a peak load of 1186 kN at 9.1 mm axial shortening, significantly outperforming its square-section counterpart CFS-03, which only achieved 847 kN at 6.1 mm. The superior performance of circular sections can be attributed to their uniform stress distribution, efficient confinement of the concrete core, and delayed onset of local buckling^37^. Circular geometry facilitates a hoop stress mechanism, enabling the steel tube to provide triaxial confinement, which enhances the ductility and post-yield strength of the composite section.

#### Performance of double-skin CFSTs (DS-CFSTs)

The interaction between the outer and inner tubes in DS-CFST specimens further amplified the composite action, as demonstrated in Fig. 6(c). Among all configurations, the CFCS-06 specimen, which featured a circular outer and inner steel tube, exhibited the highest peak load of 1325.4 kN and a maximum deformation of 12.06 mm, without significant degradation in load-carrying capacity. This indicates an effective balance of axial stiffness, energy absorption, and ductility. The inner steel tube acted not only as a core reinforcement but also provided additional lateral confinement to the concrete annulus, thereby suppressing early cracking and improving stress redistribution. The absence of premature buckling, even at high deformations, suggests that the CFCS-06 specimen achieved full utilization of the cross-sectional capacity before reaching failure^51^. Comparatively, other DS-CFST configurations such as CFSC-05 and CFSS-07 displayed earlier local instability and reduced energy dissipation capacity. The CFCC-08 specimen, which combined corrugated inner and circular outer profiles, also demonstrated favorable behaviour, with improved ductility and delayed stiffness degradation, likely due to the mechanical interlock and enhanced friction at the steel concrete interface, reducing slippage and delaying bond failure^52^. These findings confirm that circular DS-CFST configurations, particularly those with matching inner and outer profiles (e.g., CFCS-06), offer superior performance in terms of load resistance, deformation capacity, and ductility compared to square and hybrid geometries. The results strongly support the use of circular double-skin designs in applications requiring high energy absorption and post-yield stability, such as seismic columns and crash-resistant members, where controlled plastic deformation is critical for system resilience.

### Evaluation of load bearing capacity of DS-CFST with current codal provisions

#### Empirical formula

The standard formula, which is conventionally used for the load bearing capacity identification, is stress multiplied by the area as specified in Eq. (2)^18^.

2 $$\begin{aligned}\:{N}_{u,empirical}={A}_{S,t}\times\:{f}_{y}+\:{A}_{C}\times\:{f}_{c,avg}\end{aligned}$$

#### American national standard ANSI/AISC 360 − 16

The member is required to establish the limiting width-to-thickness ratio. In the present case, a width of 150 mm and a diameter of 150 mm were utilised, resulting in a width-to-thickness ratio of 75, as calculated in Eq. 3. This ratio falls within the criteria for a compact Sect.^53^.

3 $$\:{\lambda}_{p}=2.26\:\sqrt{\frac{E}{{F}_{y}}}$$

The axial compressive strength for the section is defined by the following Eq. 4.

4 $$\:{N}_{u,ANSI}=\:{F}_{y}{A}_{s}+\:{C}_{2}\:{f{\prime\:}}_{c}\:\left({A}_{c}+\:{A}_{sr}\frac{{E}_{s}}{{E}_{c}}\right)$$

$\:{C}_{2}=0.85$ for squre section and 0.95 for circular section.

#### Eurocode 4 BS EN1994-1-1:2004

From EC6, the axial compressive strength of a composite column with steel embedment can be determined as specified in Eq. 5^54^.

5 $$\:{N}_{u,\:Euro\:code\:4}={A}_{a}{f}_{yd}+0.85\:{A}_{c}\:{f}_{cd}+\:{A}_{s}{f}_{sd}$$

The application of ANSI/AISC 360 and Eurocode 4 (EC4), primarily developed for single-skin CFST members, to double-skin concrete-filled steel tube (DS-CFST) columns requires further consideration. These codes provide guidelines based on the assumption of composite action between the concrete core and the outer steel tube. However, the contribution of the inner steel tube in DS-CFST columns, which provides additional confinement and load-carrying capacity, is not explicitly addressed in these code provisions. While these provisions offer a useful starting point, they do not fully account for the additional benefits provided by the inner steel tube and the interaction between the inner and outer steel tubes in the double-skin configuration. Therefore, while the use of ANSI/AISC 360 and EC4 for DS-CFST columns is permissible for preliminary design, further refinement of these codes is required to more accurately model the behavior of DS-CFST columns.

**Table 5:** Bearing capacity comparison.

Specimens	$\:{N}_{u,\:exp}$	$\:{N}_{u,\:Empirical}$		$\:{N}_{u,\:ANSI/AISC\:360}$		$\:{N}_{u,\:EC-4\:}$	
		Strength ‘kN’	Factor	Strength ‘kN’	Factor	Strength ‘kN’	Factor*
SQT01	174.9	362.1	0.5	362.1	0.5	362.1	0.5
CIT-02	397.5	292.3	1.4	292.3	1.4	292.3	1.4
CFS-03	847.7	830.5	1.0	760.3	1.1	760.3	1.1
CFC-04	1186.8	659.9	1.8	641.5	1.9	604.7	2.0
CFSC-05	1031.6	902.8	1.1	797.7	1.3	833.4	1.2
CFCS-06	1325.4	735.9	1.8	685.5	1.9	681.6	1.9
CFSS-07	1037.1	906.6	1.1	799.7	1.3	837.2	1.2
CFCC-08	1269.7	732.2	1.7	683.4	1.9	677.8	1.9
Mean		914.2	1.3	817.8	1.5	818.8	1.6
Standard Deviation		192.4		118.2		127.4	
Coefficient of Variation (CV)		0.21		0.14		0.16	

*Factor calculated by$\:{N}_{u,\exp}$*/ predicted by Empirical*,* ANSI and EC-4*.

**Fig. 7:**
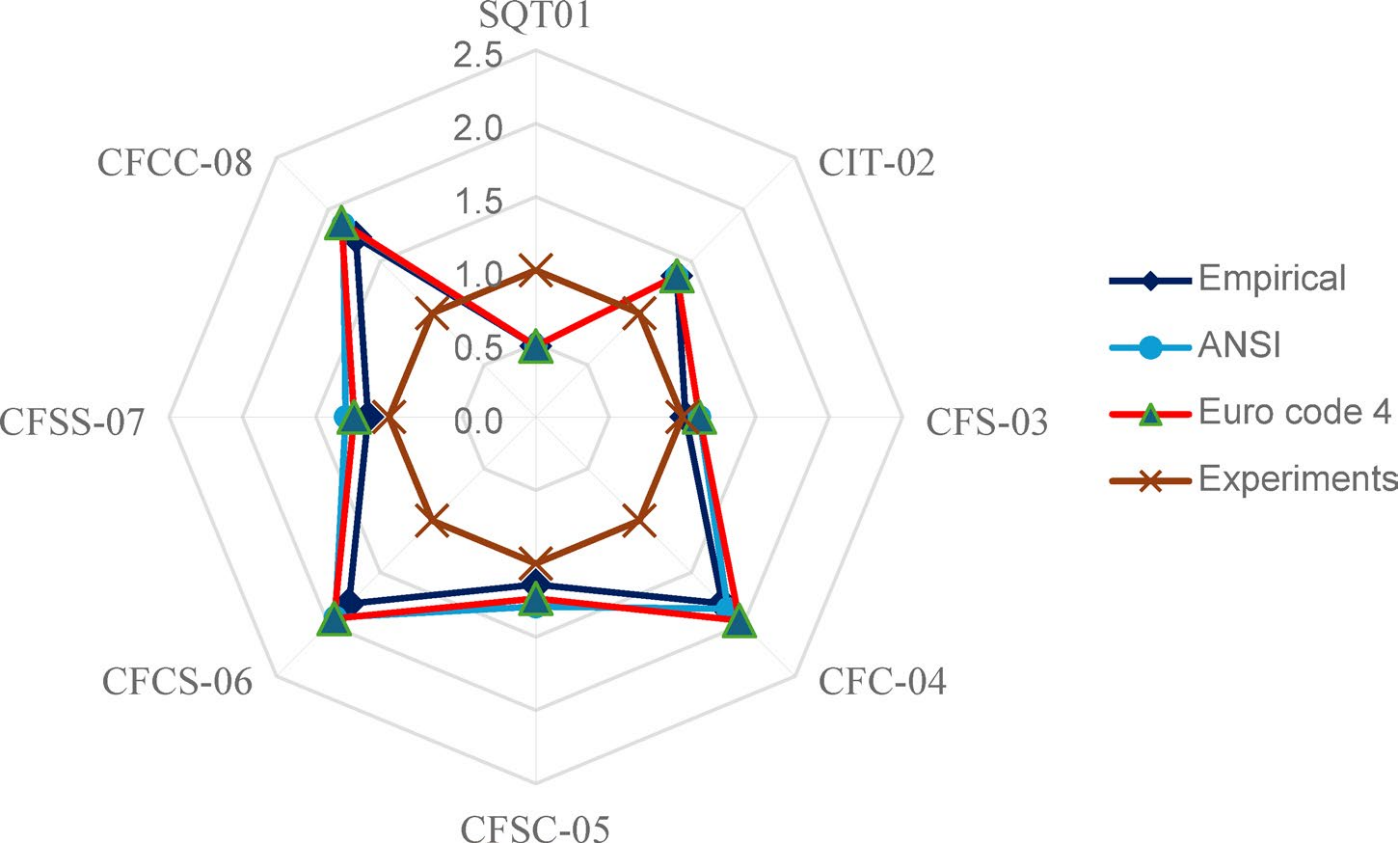
Codal comparison of the DSCFST specimens.

Figure 7 and Table 5 present a comparative analysis of the load-bearing capacities obtained experimentally and those predicted by three codal provisions Empirical, ANSI/AISC 360 − 16, and Eurocode 4 for various DS-CFST specimens. From the radar chart, it is evident that the predictions made by ANSI and Eurocode 4 closely follow similar trends. This close alignment indicates that both codes adopt a comparable approach in accounting for composite action between steel and concrete in confined columns. For double-skin CFST specimens, the experimental results consistently showed higher load-bearing capacities than those estimated by all three codes. This underestimation reflects the inherently conservative nature of current codal design practices, which prioritize safety by applying partial safety factors and simplified assumptions. While this conservatism is intended to mitigate construction variabilities and unforeseen failure mechanisms, it may also lead to underutilization of the full structural potential in well-constructed DS-CFST systems. Specimens such as CFS-03, CFSS-07, and CFSC-05 demonstrated strong agreement between the predicted and observed capacities, with strength ratios close to unity. This suggests that, for these specific geometries, the codes accurately capture the interaction between steel confinement and concrete core resistance. On the other hand, the SQT01 specimen, which is a bare square steel tube without concrete infill, showed much lower experimental strength than predicted, with a ratio of 0.5. This discrepancy can be attributed to premature local buckling, which the codal models do not fully address in the absence of composite behavior. Specimens such as CFCS-06 and CFCC-08, which incorporated less embedded steel area than CFSS-07, achieved significantly higher strength factors—up to 1.9 times the codal estimates. This highlights the benefit of circular geometry and double-skin confinement, which enhances the interaction between the steel and concrete, delays local buckling, and allows for greater energy absorption before failure. However, current design codes do not fully account for these advantages in their predictive models, leading to conservative outcomes. The analysis shows that while the ANSI/AISC 360 and Eurocode 4 provisions offer consistent and safe predictions, they may not fully reflect the performance benefits of newer DS-CFST configurations. These findings suggest the potential for updating codal methods to better accommodate advanced structural systems where geometry, confinement, and composite action contribute substantially to improved performance. The comparison indicates that both design codes provide conservative estimates of axial compressive strength for the tested CFST specimens, though variations in prediction accuracy were observed. These discrepancies can be attributed to differences in codal assumptions regarding confinement effects, composite interaction, and the treatment of local buckling in steel tubes. Eurocode 4 generally accounts for concrete confinement more explicitly, while ANSI/AISC 360 adopts simplified composite action assumptions, leading to variation in predicted capacities. Consequently, the observed deviations between experimental and analytical results highlight the need for caution in applying existing design provisions when assessing CFST members with non-conventional internal configurations.

**Table 6:** Results of ANN prediction.

Ref.	$\:{A}_{S}$, mm^2^	$\:{f}_{yi}$ MPa	$\:{f}_{y,o}$, MPa	$\:{A}_{c}$, mm^2^	$\:{f}_{ck}$, MPa	H, mm	$\:{N}_{u,exp}$	$\:{N}_{u,ANN}$
T. Ekmekyapar et al.^5^	1460.5	396.0	355.0	11649.4	42.9	343	989.1	989.1
	2670.0	396.0	535.0	10439.9	42.9	343	1943.4	1943.4
	2075.9	310.0	355.0	11034.1	42.9	343	1076.2	1076.3
	3285.4	310.0	535.0	9824.6	42.9	343	2005.0	2005.1
Zain El Abedeen et al.^9^	2317.0	284.0	326.0	23987.1	36.5	600	1672.0	1573.6
	2317.0	284.0	326.0	23987.1	37.7	600	1646.0	1590.3
	2317.0	284.0	326.0	23987.1	29.4	600	1562.0	1428.5
	2317.0	284.0	326.0	23987.1	18.0	600	1442.0	1395.0
	2317.0	284.0	326.0	23987.1	36.5	600	1662.0	1573.6
	2317.0	284.0	326.0	23987.1	37.7	600	1612.0	1590.3
	2317.0	284.0	326.0	23987.1	29.4	600	1416.0	1428.5
	2317.0	284.0	326.0	23987.1	18.0	600	1348.0	1395.0
	2317.0	284.0	326.0	23987.1	36.5	600	1602.0	1573.6
	2317.0	284.0	326.0	23987.1	37.7	600	1513.0	1590.3
	2317.0	284.0	326.0	23987.1	29.4	600	1441.0	1428.5
	2317.0	284.0	326.0	23987.1	18.0	600	1266.0	1395.0
M.F. Hassanein et al.^55^	1716.8	276.0	276.0	23717.2	38.0	432	1790.0	1789.2
Wen-Da Wang et al.^56^	2612.3	309.0	309.0	12773.7	50.0	1600	1160.0	1161.1
Pouria Ayough et al.^57^	2240.0	309.0	309.0	17360.0	50.0	1600	960.0	958.7
Serkan Tokgoz et al.^58^	2173.7	324.0	324.0	19198.0	45.0	1000	1500.0	1496.8
Junchang Ci et al.^59^	3238.1	325.0	325.0	10647.5	40.0	700	1823.0	1822.5
Junchang Ci et al.^59^	4166.8	332.0	332.0	33482.6	40.0	657	3279.0	3724.6
Junchang Ci et al.^60^	2913.1	335.0	335.0	34736.3	50.0	700	3447.0	3542.5
Zongping Chen et al.^61^	2129.7	361.0	361.0	35519.7	50.0	600	3108.0	3104.9
Zongping Chen et al.^61^	1344.4	361.0	361.0	36305.0	50.0	600	3073.0	3077.0
Muhammad Rizwan et al.^62^	2368.0	380.0	380.0	11625.0	46.0	420	1320.0	1319.4
Fa-Cheng Wang et al.^63^	4747.8	401.0	401.0	33246.2	60.0	659	2518.0	2519.1
Mizan Ahmed et al.^64^	2756.9	412.0	412.0	12629.1	40.0	400	1605.0	1604.3
CFSC-05	1419.0	310.0	310.0	21081.0	22.0	410	1031.6	1031.6
CFCS-06	1207.0	310.0	310.0	16472.0	22.0	410	1325.4	1302.8
CFSS-07	1432.0	310.0	310.0	21068.0	22.0	410	1037.1	1037.3
CFCC-08	1194.0	310.0	310.0	16485.0	22.0	410	1269.7	1292.9

#### Artificial neural network (ANN) prediction

**Fig. 8:**
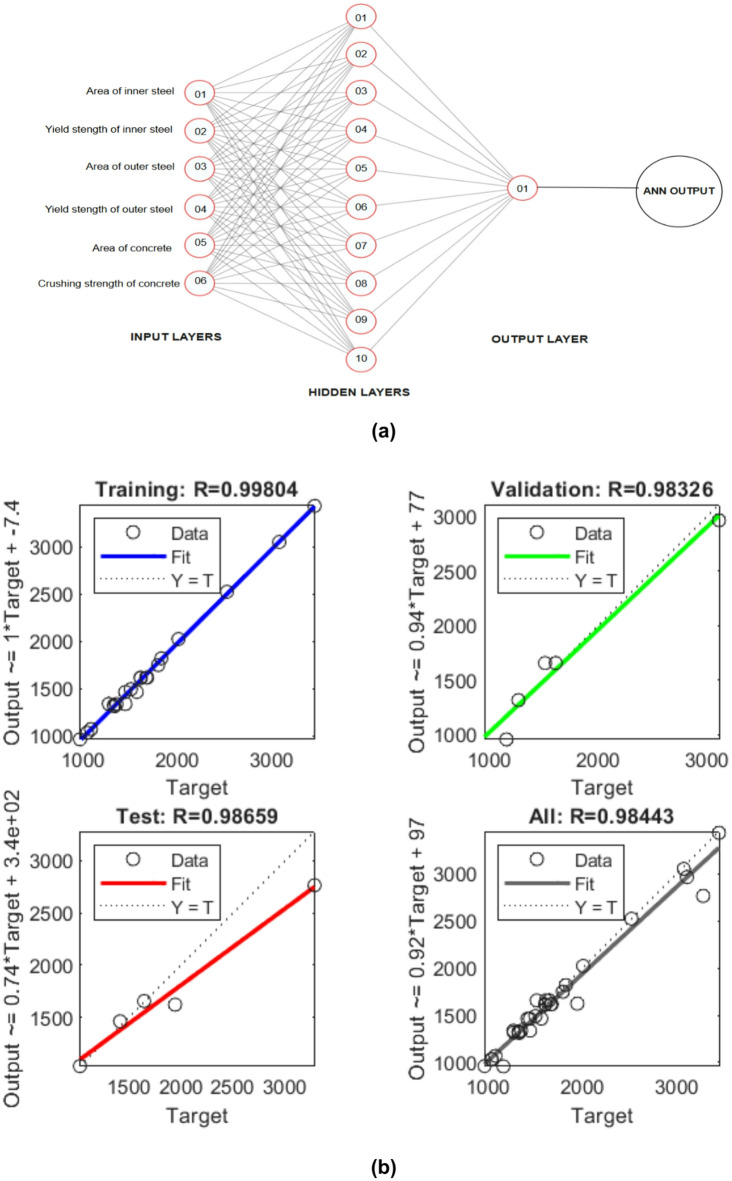
ANN model (**a**) Network architecture, (**b**) Regression analysis data prediction.

The developed Artificial Neural Network (ANN) model demonstrated high predictive capability in estimating the axial load-bearing capacity of CFST and DS-CFST columns, as substantiated by the regression plots and statistical performance metrics shown in Fig. 8. The model architecture consisted of a shallow feedforward network with 10 hidden layers, trained using the Levenberg–Marquardt optimization algorithm^65,66^. The training process used 70% of the dataset, with the remaining 30% split equally between validation and testing to prevent overfitting and ensure generalizability. Table 6 presents the input parameters that included key structural features, such as the inner and outer steel areas (A_s_), the concrete area (A_c_), steel yield strength (f_y_), concrete compressive strength (f_ck_), and the column height (H). The model was trained on data from 20 specimens, with each specimen characterized by six input parameters, and yielding 20 corresponding axial load-bearing capacities (N_u, exp_) from experimental measurements. While the limited dataset constrained the model, ANN’s capability to capture complex, non-linear relationships in small datasets made it a suitable tool for preliminary predictive modeling.

However, we acknowledge the reviewer’s concern regarding the small number of specimens used in the modeling (20 specimens). In response, we recognize that including more data points, particularly for variations in geometric and material configurations such as D/t and H/D ratios, would improve the robustness of the model. To address this, we incorporated benchmark data from previous studies (T. Ekmekyapar et al., Zain El Abedeen et al., etc.) to expand the model’s training set and ensure its generalizability across a wider range of geometries and materials.

The ANN model achieved a high coefficient of determination (R²) value of 0.9969, indicating strong agreement between the experimental values and predicted outputs. This performance was consistent across training (*R* = 0.992), validation (*R* = 0.9817), and testing (*R* = 0.9993) datasets, as reflected in the regression plots^67,68^. Notably, the “All Data” regression yielded *R* = 0.9803, reaffirming the model’s reliability. The comparison with benchmark data, such as T. Ekmekyapar et al. and Zain El Abedeen et al., ^69–71^ validated the generalizability of the ANN model, showing consistent results with experimental observations. In the case of CFCS-06, with an experimental axial capacity of 1325.4 kN, the predicted value was 1302.8 kN, exhibiting a deviation of less than 2%.

Although the current dataset is small, the ANN framework, when combined with proper input parameters and optimization strategies, presents a promising tool for predicting axial strength in CFST and DS-CFST columns. Further refinement with additional data from finite element (FE) verified models and a larger number of specimens would further enhance the model’s accuracy and make it more reliable for predicting the axial strength of such columns. We have included an error histogram that indicates minimal deviation between target and predicted values, further confirming the model’s precision and capability^72^. These findings demonstrate that, while the ANN model was built on a limited dataset, it can serve as a potent and efficient alternative to traditional analytical models for predicting the axial strength of CFST/DS-CFST columns.

## Conclusion

The axial compressive performance of double-skin concrete-filled steel tube short columns with solid core concrete infill was found to vary between square and circular geometries for the inner and outer steel tubes. The experimental findings and the predictive results are discussed in this chapter.

i. The axial compressive capacity of the CFS-03 specimen was 384% greater than the non-infilled square tube SQT-01, and the CFC-04 exhibited 198% higher performance compared to the CIT-02 specimen. However, the SQT specimen failed earlier than the empirical predictions, suggesting that different factors influence the behaviour of square geometry members.
ii. The double-skin specimens consistently demonstrated superior performance to the single-skin CFS and CFC, exhibiting approximately 56% higher strength than the CFS-03 specimen.
iii. Regarding the geometrical configuration, the circular inner and outer profiles outperformed the square geometry. The radial stress distribution in the circular profile led to uniform stress distribution and more distinct failure modes. Furthermore, the complete solid core concrete absorbed more energy and mitigated the internal bursting effect in the tubes.
iv. Local buckling was observed in the SQT-01 and CIT-02 specimens, while the square outer geometry specimens displayed outward buckling and elephant foot buckling. Conversely, the outer circular geometry specimens, CFC-04, CFCS-06, and CFCC-08, experienced radial elongation and crushing failure.
v. The predictive models based on empirical methods showed closer correlation with the experimental results for the DS-CFST specimens, while the ANSI/AISC-360 and Eurocode 4 design approaches provided conservative estimates. The ANN prediction model yielded highly accurate results (R² = 0.99) with reduced training epochs. However, it should be noted that the ANN model was developed using a limited dataset (20 specimens) and is therefore applicable only within the investigated range of geometric and material parameters. A larger experimental database would be required to generalize the model for broader design applications.
vi. The CFCS-06 specimen, featuring a circular outer tube and a square inner tube, demonstrated approximately 27.8% higher axial compressive strength, stiffness, and lower deformation compared to the CFSS-07 specimen, which has both a square outer and inner tube. This enhanced performance is attributed to the more efficient confinement and stress distribution provided by the circular outer tube, which plays a crucial role in improving the overall structural behavior of the column.Credit authorship contribution statement.

## Data Availability

The datasets used and/or analysed during the current study are available from the corresponding author on reasonable request.

## Abbreviations

CFST: Concrete-filled steel tube
DS-CFST: Double-skinned solid concrete-filled steel tube
SQT: Square steel tube
CIT: Circular tube
CFS: Concrete-filled square tube
CFC: Concrete-filled circular tube
CFSC: Concrete-filled square outer and circular inner tube
CFCS: Concrete-filled circular outer and square inner tube
CFSS: Concrete-filled outer square and inner square tube
CFCC: Concrete-filled outer circle and inner circle tube
λ: Slenderness ratio
${\boldsymbol{A}}_{\boldsymbol{s},\boldsymbol{o}}$: Area of outer steel tube, mm^2^
${\boldsymbol{A}}_{\boldsymbol{s},\boldsymbol{i}}$: Area of outer steel tube, mm^2^
${\boldsymbol{A}}_{\boldsymbol{s},\boldsymbol{t}},\:{\boldsymbol{A}}_{\boldsymbol{s}\boldsymbol{r}}$: Area of outer steel tube, mm^2^
${\boldsymbol{A}}_{\boldsymbol{s},\boldsymbol{\%}}$: Area of outer steel tube, mm^2^
${\boldsymbol{B}}_{\boldsymbol{o}}$: Width or diameter of the outer tube, mm
${\boldsymbol{B}}_{\boldsymbol{i}}$: Width or diameter of the inner tube, mm
H: Height of the column, mm
${\boldsymbol{t}}_{\boldsymbol{o}} \:{\boldsymbol{t}}_{\boldsymbol{i}}$: Thickness of the outer, inner tube, mm
${\boldsymbol{f}}_{\boldsymbol{c}},\:{\boldsymbol{f}\boldsymbol{{\prime\:}}}_{\boldsymbol{c}},\:{\boldsymbol{f}}_{\boldsymbol{c}\boldsymbol{d}}$: Compressive strength of concrete, MPa
${\boldsymbol{f}}_{\boldsymbol{c},\boldsymbol{a}\boldsymbol{v}\boldsymbol{g}}$: Average compressive strength of concrete, MPa
${\boldsymbol{\lambda\:}}_{\boldsymbol{p}\:}$: Non-dimensional parameter, ANSI
${\boldsymbol{E}}_{\boldsymbol{s}},{\boldsymbol{E}}_{\boldsymbol{c}}$: Youngs modulus of steel and concrete, MPa
${\boldsymbol{f}}_{\boldsymbol{y}},\:{\boldsymbol{f}}_{\boldsymbol{y}\boldsymbol{d}}$: Yield strength of steel tube, MPa
${\boldsymbol{C}}_{2}$: Reduction factor, ANSI
DI: Ductility index
*K*: Stiffness
${\varDelta\:}_{\boldsymbol{u},\:0.85}$: Deflection 85% of ultimate load
${\varDelta\:}_{\boldsymbol{u}}$: Deflection at ultimate load
${\varDelta\:}_{\boldsymbol{y}}$: Deflection at yield load
${\boldsymbol{N}}_{\boldsymbol{u},\mathbf{e}\mathbf{x}\mathbf{p}}$: Ultimate compressive load by experiment, kN
${\boldsymbol{N}}_{\boldsymbol{y},\boldsymbol{e}\boldsymbol{x}\boldsymbol{p}}$: Yield compressive load by experiment, kN
${\boldsymbol{N}}_{\boldsymbol{u},\boldsymbol{e}\boldsymbol{m}\boldsymbol{p}\boldsymbol{i}\boldsymbol{r}\boldsymbol{i}\boldsymbol{c}\boldsymbol{a}\boldsymbol{l}}$: Ultimate load predicted by empirical, kN
${\boldsymbol{N}}_{\boldsymbol{u},\boldsymbol{A}\boldsymbol{N}\boldsymbol{S}\boldsymbol{I}/\boldsymbol{A}\boldsymbol{I}\boldsymbol{S}\boldsymbol{C}\:\:360}$: Ultimate load predicted by ANSI, kN
${\boldsymbol{N}}_{\boldsymbol{u},\:\boldsymbol{E}\boldsymbol{C}4}$: Ultimate load predicted by Euro standard, kN
${\boldsymbol{N}}_{\boldsymbol{u},\boldsymbol{e}\boldsymbol{x}\boldsymbol{p}}$: Ultimate load predicted by ANN, kN.
